# Genomic positions of co-expressed genes: echoes of chromosome organisation in gene expression data

**DOI:** 10.1186/1756-0500-6-229

**Published:** 2013-06-13

**Authors:** Teresa Szczepińska, Krzysztof Pawłowski

**Affiliations:** 1 Nencki Institute of Experimental Biology, PAS, Pasteura 3, Warsaw 02-093, Poland; 2 Warsaw University of Life Sciences, Nowoursynowska 166, Warsaw 02-787, Poland; 3 Institute of Genetics and Biotechnology, Faculty of Biology, University of Warsaw, Pawinskiego 5a, Warsaw 02-106, Poland; 4 Institute of Biochemistry and Biophysics, Polish Academy of Sciences, Pawinskiego 5a, Warsaw 02-106, Poland

**Keywords:** Chromosome territory, Gene expression, Cluster analysis, Nuclear structure

## Abstract

**Background:**

The relationships between gene expression and nuclear structure, chromosome territories in particular, are currently being elucidated experimentally. Each chromosome occupies an individual, spatially-limited space with a preferential position relative to the nuclear centre that may be specific to the cell and tissue type. We sought to discover whether patterns in gene expression databases might exist that would mirror prevailing or recurring nuclear structure patterns, chromosome territory interactions in particular.

**Results:**

We used human gene expression datasets, both from a tissue expression atlas and from a large set including diverse types of perturbations. We identified groups of positional gene clusters over-represented in gene expression clusters. We show that some pairs of chromosomes and pairs of 10 Mbp long chromosome regions are significantly enriched in the expression clusters. The functions of genes involved in inter-chromosome co-expression relationships are non-random and predominantly related to cell-cell communication and reaction to external stimuli.

**Conclusions:**

We suggest that inter-chromosomal gene co-expression can be interpreted in the context of nuclear structure, and that even expression datasets that include very diverse conditions and cell types show consistent relationships.

## Background

Ever since genome-wide gene expression datasets became available, regularities and similarities in gene expression profiles have attracted attention [1]. Although typical operons are a feature characteristic of bacteria, operons or operon-like gene groups are found in many eukaryotic lineages [2]. More generally, several studies have shown co-expression of neighbouring genes in eukaryotes, from yeast to humans [3,4]. These positionally co-expressed genes included tissue-specific genes [5,6] and house-keeping genes [7]; however, as reviewed by Hurst and colleagues [8], it is a general phenomenon. It has also been established that positionally-clustered co-expression units are conserved in mammalian evolution [9]. The first genome-scale report of the genomic clustering of co-expressed genes in humans came from the “transcriptome map” by Versteeg and colleagues [10]. It showed that many human genes that are highly expressed were clustered in genomic domains (“ridges”), 5 – 15 Mbp wide. Ridges were gene-rich and contained both housekeeping genes and highly expressed genes, active only in certain tissues [11]. In contrast to ridges, there are gene-poor regions of similar size enriched with genes that have low expression [12]. Fluorescent in situ hybridization (FISH) experiments demonstrate that ridges are in general located closer to the nuclear centre than anti-ridges [12].

Various reasons for local gene co-expression in eukaryotes have been identified, including the presence of close paralogues and the existence of bi-directional promoters [3]. Yet, even after allowing for these factors, local co-expression remains and chromatin structure is suspected to be an important factor [8]. A recent analysis [13] suggests that the “human gene co-expression landscape” is functionally relevant and includes house-keeping genes, tissue-specific genes, and specific pathways.

The three-dimensional organization of chromosomes in the human interphase nucleus is relevant for gene regulation, yet it is far from being fully understood. Chromosomes can be divided into domains of open chromatin, where genes are preferentially expressed, and domains of closed chromatin, where they are not [5]. In the eukaryotic nucleus, each chromosome is confined to a discrete region called a chromosome territory [14]. For a long time there has been a debate as to whether chromosomes are separate or intermingled [15-17]. This has recently been resolved as a result of several elegant high-throughput studies elucidating nuclear structure, as exemplified by Noble and co-workers [18] who succeeded in building a three-dimensional model of the yeast genome. The validity of the chromosomal territory concept has also been recently demonstrated by Dekker and co-workers who mapped long-range chromosomal interactions in two human cell types [19]. Also, a trend for specific inter-chromosomal associations between co-regulated genes in human erythroid cells has been reported [20]. Areas of intermingling enable interchromosomal interactions and may imply interchromosomal rearrangements. Such intertwining of specific chromosome pairs in human lymphocytes correlates with the chromosome translocation frequency in those cells [21]. The presence of “transcription factories”, i.e. regions of enhanced transcriptional activity, [20,22-24] in the intertwined regions and the effect of changed transcription on the inter-chromosomal interactions suggests that these events strongly influence chromosome organization in mammalian cells [21]. Furthermore, interchromosomal interactions can occur via extended chromatin loops. Such contacts between different chromosomal loci are called chromosome kissing [25,26]. Some of these contacts may occur because of preferred chromosome neighbourhoods and because of the transcriptional machineries shared, others may be related to specific regulatory functions [25]. Kissing events have been shown to be involved in both gene silencing and gene enhancing [27].

Chromosome territories (CT) may occupy preferred sub-nuclear positions and have a complex three-dimensional shape [28]. Their positioning is non-random and heterologous CT groupings are favoured [28]. Regional gene density has been suggested to be the decisive parameter determining the radial positioning of chromatin in the human nucleus [29]. Although some chromosome arrangement principles hold over different cell lines [12], tissue-specific organization of chromosomes has been shown in mouse cells [30]. Small groups of chromosomes do form various types of spatial clusters in different tissues; also, relative distances between pairs of chromosomes depend on the tissue [30]. A recently demonstrated mechanism of genome reorganization within the nucleus involves the movement of chromosomal regions relative to the nuclear lamina during differentiation of embryonic stem cells [31]. This reorganization is related to activation of transcription [31].

There are reports that a higher order organization of genes between and within chromosomes is constrained by transcriptional regulation in *Saccharomyces cerevisiae*[32]. Results of a transcriptional regulatory network analysis of this organism illustrate that a majority of the transcription factors tend to preferentially regulate their targets on one or only a few chromosomes. Several transcription factors have a strong preference for regulating genes in specific regions on the chromosomal arms, and most transcription factors tend to prefer to bind targets clustered positionally within a specific chromosome region [32]. It has been suggested recently that three-dimensional organisation preferences may even be conserved in evolution [33].

While the patterns of the three-dimensional organization of the chromosomes in the nucleus are not solved, the involvement of several chromosome structure features influencing the organisation (e.g. gene density or chromosome size) has been suggested. Assuming constrained positions of chromosomes and the influence of chromosome - chromosome interactions on gene expression, we were looking for patterns of chromosome position within groups of genes with similar expression patterns. More than a decade ago, Cohen et al. introduced chromosome correlation maps [3]. We follow in a similar spirit. Recently, also Woo et al. studied expression correlation in several genomic datasets, human and mouse [34]. They found pervasive co-expression, both local and long-range. Our approach differs from that of Woo et al. by not focusing on correlations, but rather on expression clusters and the presence of pairs and larger groups of distant genomic regions (also inter-chromosomal ones) within the clusters. Also, we strived to find functional significance in the observed long-range co-expression.

Gene expression microarrays offer a powerful technique for the exploration of the molecular biology of the cell [1]. For example, gene expression clustering has been used for classification and clinical outcome prediction in disease [35] or for elucidation of functional and regulatory gene modules [36]. In this study, we have analysed two large and diverse tissue microarray gene expression datasets using two different clustering methods. First, we found groups of positional clusters appearing more often than could be deemed random within expression clusters. Second, we found an enhanced presence of specific chromosome pairs and chromosome region pairs in the expression clusters. Third, we analysed functional properties of inter-chromosomal region pairs enriched in the expression clusters.

## Results

The aim of this study was to explore relationships between human gene expression and gene position in the genome. We analysed two types of gene expression microarray data of different origins. The datasets included many different human tissues (see Methods). The analyses involved three main steps:

1. Defining gene co-expression clusters to be used in further study – groups of genes with similar expression profiles (expression patterns across sets of samples).

2. Finding patterns in genome positions for genes belonging to such co-expression clusters:

a) Finding groups of positional clusters within co-expression clusters. We examined whether genes from one co-expression cluster form positional clusters in the genome. In addition, we examined whether there are pairs or groups of such positional clusters within a co-expression cluster.

a) Finding pairs of genome regions (for whole chromosomes and for 10 Mbp-wide regions) that contain more genes belonging to the same co-expression cluster than expected by chance.

3. Analyses of functional annotation enrichment for groups of co-expressed genes from particular genomic regions.

### Gene co-expression clusters

Genes belonging to one co-expression cluster exhibit similar expression profiles. Expression profiles may involve many tissues and many experimental conditions. We decided to use tissue-wide expression profiles in order to obtain functionally relevant groupings of genes that function together, so called transcription modules. Keeping in mind that nucleus architecture may differ between tissues, by selecting profiles across many tissues, we focused on patterns that are more likely not to be tissue-specific. We analysed two data sets of different origins. A clustering method suited to each data type was chosen. The NeMo data [37] is a collection of microarray data sets from different experiments involving various types of perturbations. That includes various tissues and cell lines, diseases, chemical treatments, chemical exposure levels. The graph-based clustering method employed by Yan et al. [37] enables selection of transcription modules from such a dataset. NeMo transcription modules are groups of genes that form co-expression clusters in multiple datasets generated under different conditions. The second dataset, the Symatlas dataset, is an atlas of measurements from different tissues performed in one broad experiment [38]. To calculate expression correlations, tissue-wide profiles are taken into account. We used hierarchical clustering with correlation as a distance measure to obtain co-expression clusters from this data. The sizes of gene co-expression clusters obtained using both clustering methods are similar. Clusters obtained by the two different approaches differed (a) in the total number of clusters, (b) in the number of genes in all clusters considered together and (c) in the fact that clusters from NeMo data overlap in contrast to the clusters from Symatlas data. Thus, there are 222 clusters containing together 2578 genes for Symatlas data and 4727 clusters containing together 716 genes for NeMo data (see Methods). These two datasets and the different clustering methods used resulted in finding potential transcription modules of various structures.

### Finding patterns in genome positions for genes belonging to such co-expression clusters: the genomic positions of genes from the same co-expression cluster are not random

**Figure 1:**
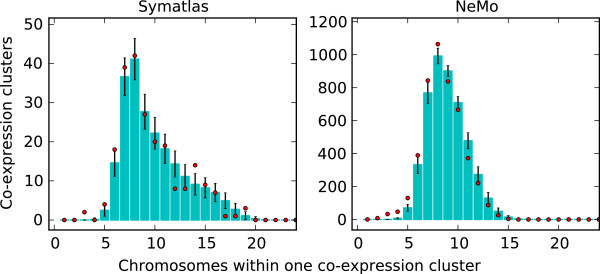
**The numbers of chromosomes within co-expression clusters.** For example, in Symatlas data, the most common number of chromosomes represented in an expression cluster is eight, and there are more than 40 such expression clusters. Dots - Symatlas (left) and NeMo (right) data. Bars - randomised data with standard deviation (SD) shown (see Methods).

**Figure 2:**
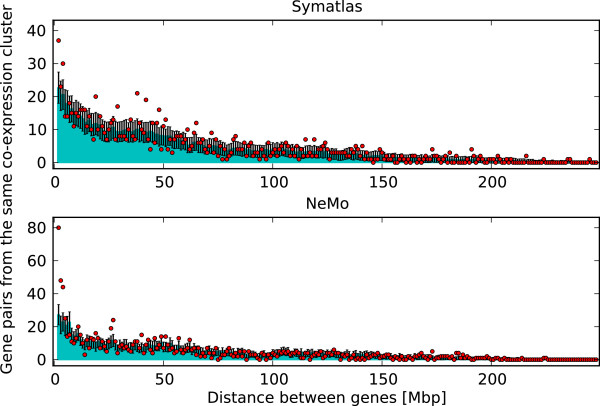
**Distances in sequence between genes from the same co-expression cluster.** Dots - Symatlas (top) and NeMo (bottom) data. Bars - randomised data with SD shown. The number of inter-chromosomal gene pairs within one co-expression cluster is 163 (compared to an average 26.81 + - 5.26 in randomised data) and 214 (compared to an average 23.42 + - 5.28 in randomised data) for Symatlas – and NeMo data, respectively.

The existence of chromosomal territories, as described in the literature, prevents interaction of many chromosomes together at the same time. Only genes from a few chromosomes can be in close proximity at the same time. Although long loops with active or inactive genes are also observed, they have not been recognised as a massive event. We analysed the number of chromosomes represented in one co-expression cluster. The observed numbers are lower than in the situation when gene positions are assigned randomly (see Figure 1), albeit this trend does not reach significance. The low number of chromosomes in one co-expression cluster is partly the result of the tendency of co-expressed genes to be positioned in close proximity on a chromosome. Such positional gene clusters have been observed in different organisms and with a gene expression similarity calculated in many different ways [2]. For this study, the number of positional clusters within co-expression clusters in both datasets studied is significantly higher than when gene positions are assigned randomly. In Symatlas data, there are 71 positional clusters, while with randomised positions the average number is 28.5 (significant difference, permutation test p-value below 10^-4^). In NeMo data this number is 173, while the average for randomized positions equals 47.2 (p-value below 10^-4^). The tendency of co-expressed genes to be in proximity can also be observed without applying definitions of positional clusters. As can be expected from the literature [2], the distances between the positions of genes within one co-expression cluster are lower than in a randomised situation, or pairs of nearby genes occur in the expression clusters more often than expected by chance (see Figure 2).

### Finding groups of positional clusters within co-expression clusters that occur more often than expected by chance

Besides looking at positionally clustered genes in a single chromosome region, we checked to determine if there was more than one such positional cluster within one co-expression cluster. A group of positional clusters was defined as two or more positional clusters that could belong to different chromosomes or to distant regions of the same chromosome. In all co-expression clusters, the number of groups of positional clusters was 17 and 108 for Symatlas and NeMo, respectively. This is significantly more than for randomised gene positions (see Methods): averages 2.7 (p-value below 10^-4^) and 19.2 (p-value below 10^-4^) for Symatlas and NeMo data, respectively. We also checked to see if the higher than random numbers of groups of positional clusters within one co-expression cluster were the result of a higher than expected number of positional clusters in general. For this comparison, the assignment of each positional cluster to a co-expression cluster was randomised (see Methods). The number of groups of positional clusters in non-randomised data is higher in comparison to cases of positional clusters spread among co-expression clusters randomly, namely for Symatlas: 17 (real data) vs 9.9 (randomised), p-value 0.002 and for NeMo data: 108 vs 91.1, p-value 0.01. We observe a significantly higher-than-expected number not only of positional gene clusters but also of groups of such positional clusters within one co-expression cluster.

### The structure of groups of positional clusters

**Figure 3:**
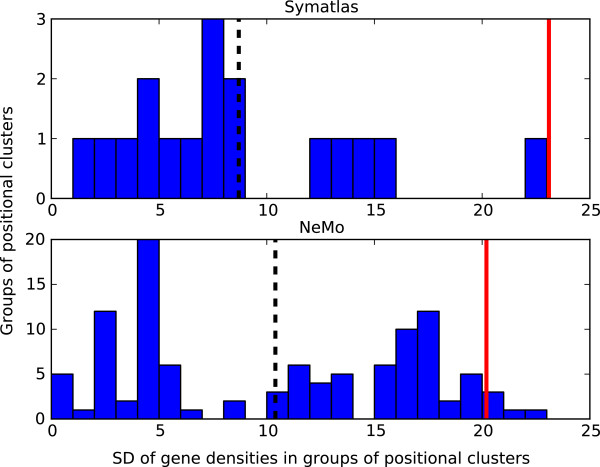
**Standard deviations of gene densities [genes/Mbp] within groups of positional clusters.** Groups of positional clusters are defined as positional clusters belonging to the same co-expression cluster. SD was calculated for gene densities from each group of positional clusters separately (dashed lines indicate the mean SD) and for gene densities from all positional clusters together (red lines). Symatlas (top) and NeMo (bottom) data.

**Figure 4:**
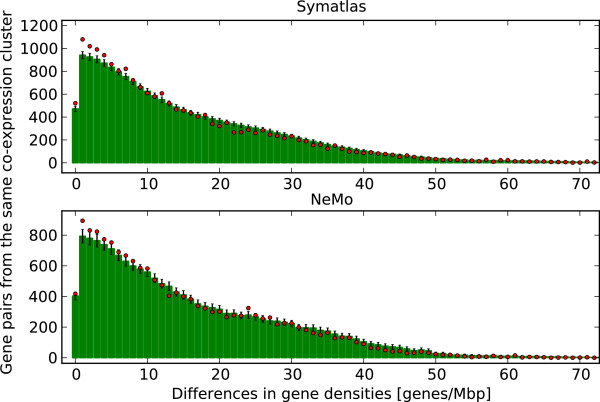
**Differences in gene densities between genes from the same co-expression cluster and from different chromosomes.** Dots - Symatlas (top) and NeMo (bottom) data. Bars - randomised data with SD shown.

The average number of genes in a positional cluster is 2.4 (standard deviation, SD 0.7) and 2.5 (SD 0.8), in Symatlas data and in NeMo data, respectively. The average number of positional clusters within one co-expression cluster is 2.3 (SD 0.9) in Symatlas data and 2.5 (SD 1.1) in NeMo data. These numbers are low. The results of this analysis indicate clusters with few genes rather than large regions of the genome that contain many genes. Positional clusters are found in regions with varied local gene density. We checked to see if positional clusters from one co-expression cluster are characterised by similar gene density. Densities of genes within one group of positional clusters from the same co-expression cluster were compared to gene densities from all groups of positional clusters considered together. For each group of positional clusters, the standard deviation of gene densities (counted in genes per 1 Mbp) was calculated and the mean of all standard deviations was taken. The mean standard deviation is 8.7 (SD 5.3, Symatlas data) and 10.4 (SD 6.6, NeMo data). The average and standard deviation of gene densities from all positional cluster groups considered together was calculated as well. The average gene density for Symatlas data is 35.6 (SD 23.1, values ranging from 6 to 114); and for NeMo data it is 29.2 (SD 20.2, values from 5 to 80). The mean standard deviation for all groups relative to the standard deviation of all gene densities taken together is relatively low, 38% (8.7 vs. 23.1) and 51% (10.4 vs. 20.2) for Symatlas and NeMo data, respectively (see dotted and red lines in Figure 3). Genes from the same co-expression cluster show gene-density similarity. The differences of gene densities for pairs of genes from the same co-expression cluster were compared to such differences in a randomised situation, i.e. with gene positions assigned randomly (see Methods). The lower differences in gene-density are observed not only when we take into account all gene pairs from the same co-expression cluster but also when we count only those pairs that contain genes from different chromosomes. Gene pairs from the same co-expression cluster but from different chromosomes usually have similar local gene densities compared to pairs of genes with randomised positions (see Figure 4). Thus, the tendency of similarity in gene density of co-expressed genes is observed. It is not related to similarity in the gene density of genes located close to each other on the chromosome sequence.

### Finding pairs of genome regions: genes from some regions in the genome are often strikingly co-expressed

**Figure 5:**
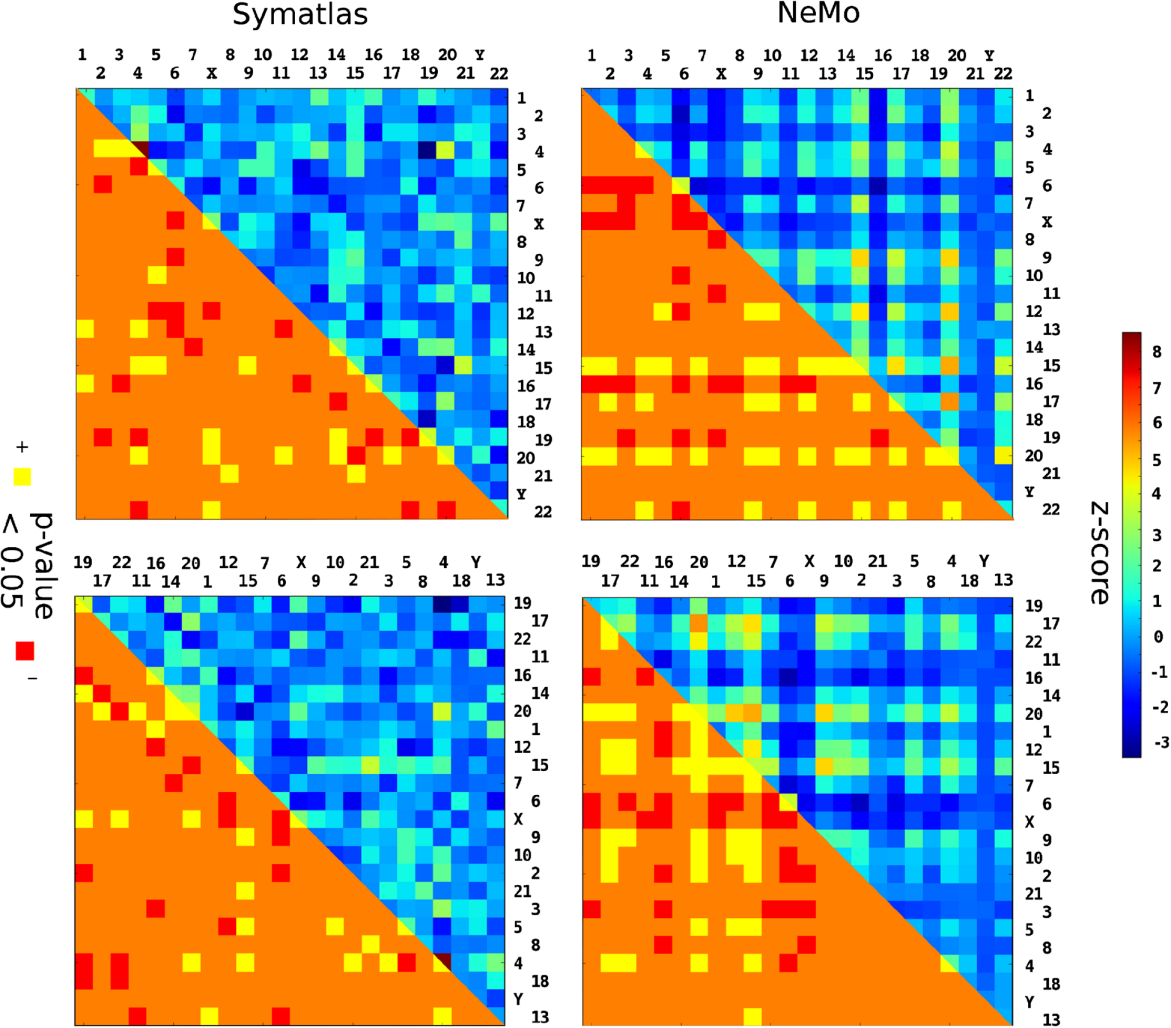
**Chromosome pairs from which genes are found together in one co-expression cluster.** Square colours correspond to the frequency of observation of genes from a pair of chromosomes within the same co-expression cluster. The upper triangle colours indicate the number of standard deviations between co-expression gene pair counts for real and randomised data, the lower triangle – p-value (0.05 threshold for each one-tailed test). Chromosome order is by chromosome size (top) or chromosome gene density (bottom). Symatlas data (left), NeMo data (right).

We also determined if some chromosomes are represented in one co-expression cluster more often than expected. For each pair of chromosomes, we counted pairs of genes that belong to those chromosomes and are found in one co-expression cluster. To obtain a reference, we repeated the procedure for the same clusters but with randomised gene positions and compared the counts. In both datasets there are pairs of chromosomes that often appear in one co-expression cluster (Table 1). These pairs depend on expression modules extracted from the datasets; hence, they are not identical for both datasets (see Figure 5). Significance of a pair of chromosomes co-occurring in co-expression clusters was estimated by Z-score calculation and permutation tests, whereby gene positions were randomised (see Methods). Here, “appearance” means observing a pair of chromosomes with significance that is more than 3 standard deviations. The number of chromosomes that frequently appear with a given chromosome varied between chromosomes. Some chromosomes have many such partner chromosomes that they often appear with. In the NeMo dataset, chromosomes 15, 20 and 17 have the largest number of partner chromosomes, while in the Symatlas dataset none of the chromosomes are distinct (see Figure 5). Some groups of genes associated with particular chromosomes are co-expressed with each other. We found those groups by hierarchical clustering with the matrix of chromosome pair co-occurrence significance used as the similarity matrix (see Methods). Chromosomes 9, 12, 15, 17, 20, 22 are grouped together in the NeMo data. In the Symatlas dataset, there are two groups: chromosome 15 with 21 and chromosome 4 with 20 (Additional file 1). Both chromosomes 17 and 22 have high overall gene density (see Additional file 2). Chromosomes 20, 12 and 15 have a medium overall gene density. Chromosomes 15 and 21 are achrocentric chromosomes, i.e. their short arms contain rDNA that is a part of the nucleolus (see Additional file 2).

**Table 1:** Pairs of chromosomes that often appear in one co-expression cluster

NeMo	17-20, 15-20, 12-20, 9-15, 9-20, 15-17, 12-15, 20-22, 15-22, 6-6, 9-17, 4-20, 15-15, 7-15, 12-17, 4-15, 10-15, 17-22
Symatlas	4-4, 20-20,4-20,19-19, 15-21,16-16

The difference between the number of occurrences in non-randomised and randomised data is at least 3 standard deviations. The pairs of chromosomes are presented in order of descending significance.

**Figure 6:**
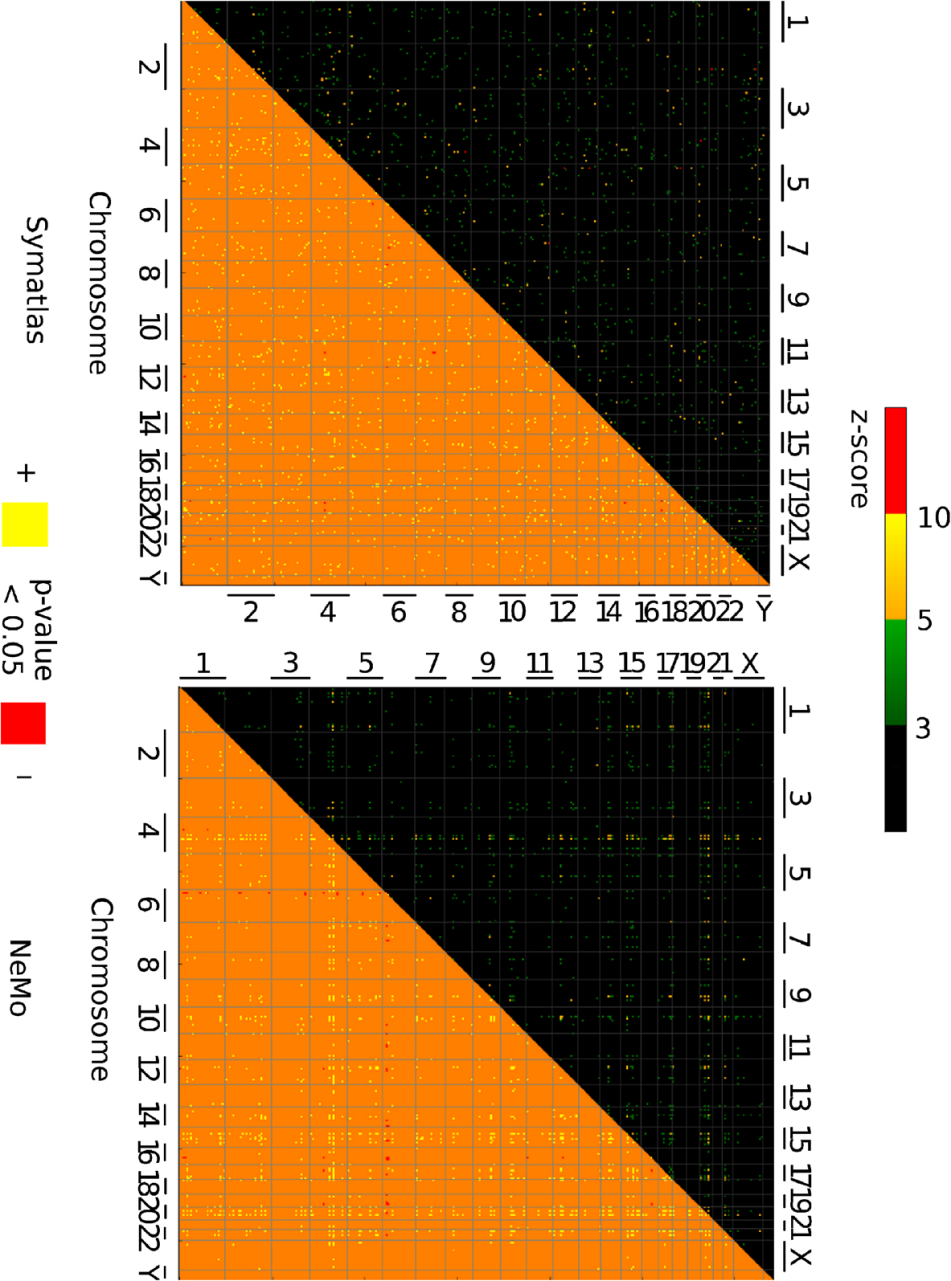
**Ten Mbp-wide regions in the genome from which genes are found together in one co-expression cluster.** Square colours correspond to the frequency of observation of genes from a pair of genomic regions within the same co-expression cluster The upper triangle colours indicate the number of standard deviations between co-expression gene pair counts for real and randomised data (cut-off 3, 5 or 10 standard deviations), the lower triangle – p-value (0.05 threshold for each one-tailed test). Symatlas data (top), NeMo data (bottom).

Besides taking into account whole chromosomes, we checked if there are chromosome regions associated with genes that are often found together in one co-expression cluster. The chromosomes were divided into 10 Mbp regions of fixed length. The procedure for assessing significant pairs of regions with co-expressed genes was the same as for the whole chromosomes. Indeed, there are pairs of regions from which genes are significantly often co-expressed, called here partnering regions (see Figure 6). Partnering regions are those pairs of regions that have co-expression significance above a cut-off, 3 or 5 standard deviations. These are both regions from the same chromosome, but distant in sequence, and regions from different chromosomes. For example, there are striking “smudges” of such regions for the NeMo dataset and chromosomes 4, 6 and 20. Interestingly, there is a wide variation between the numbers of partner regions for a single genomic region. In both datasets, we see some prominent regions that have many partners from different chromosomes. These regions are not identical for both datasets and are easily visible only for NeMo data (see Figure 6). We looked into “focus regions” that have the largest number of partnering regions (constituting around 4% of all 10 Mbp genomic regions defined in this study). Those regions together with their chromosome positions can be found in Additional file 3. Only a few chromosomes have such regions in both datasets. Among them, chromosomes 4 and 20 appear particularly interesting. In both datasets studied, they have significant partnering regions that are not the same but are close to each other. Also, region 233 from chromosome 14 is a region that has significant partnering regions in both datasets. To better understand cases of regions having many partner regions, a closer investigation of regions 81 - 89 from chromosome 4, region 233 from chromosome 14 and regions 283 - 287 from chromosome 20 was performed.

### Analyses of functional annotation enrichment

In order to gain an insight into the possible functional role of the co-expressed region pairs, for each of these “focus regions”, its genes together with genes from partnering regions found in the dataset were used as queries in functional term enrichment analysis (see Methods). Genes from Symatlas data from region 87 and its partnering regions are most significantly enriched in functional annotation terms: plasma and extracellular space (resulting from the presence of genes such as serpins, apolipoproteins, and fibrinogens). Also, genes from Symatlas data from region 285 are most significantly enriched in the term extracellular space resulting from the presence of genes such as cystatins. Groups of genes related to the extracellular annotation term for regions 87 and 285 and their partner regions do not overlap (see Additional file 4). Genes from NeMo data from regions 82, 84, 89, 233, 283, 285, 287 and their partner regions (each region together with its partnering regions is analysed separately) are enriched by the cell cycle phase functional annotation term and related terms (see Additional file 4).

Further, genes from all significant pairs of regions (see Figure 6) were used together as one query gene set in functional term enrichment analysis. Thus, queries included genes from regions which significantly often appear together in one co-expression cluster. Cut-offs of 3, 5 or 10 standard deviations were applied (see Additional file 5). The most significant functional annotation terms related to Symatlas genes are the extracellular region, signalling, secreted, and for the highest significance cut-off also chemokine activity. Most significant terms related to NeMo genes are related to cell cycle, chromosome, histone, also the MHC protein complex and the immunoglobulin C1-set domain.

Besides the analysis of pairs of expression-correlated regions, functional analysis of groups of positional clusters located in the same co-expression cluster was performed. The query gene set included genes from all such groups of positional clusters taken together. Not surprisingly, the same functional terms appeared that had previously been recognised for significant pairs of chromosome regions. In the NeMo dataset, we see functional terms related to immunological response due to the presence in the co-expressed groups of positional clusters of human leukocyte antigen (HLA), collagen, complement component (C1R, C1S), and chemokine (CXCL9, CXCL11) genes. In the Symatlas dataset, functional terms related to the HLA complex and the group of cytochromes (‘hydroxylation of lipid’, ‘biosynthesis of steroid hormone’ terms) are recognised. Also, terms such as extracellular, signal, secreted are also present for the NeMo dataset (see Additional file 6).

## Discussion

It has to be borne in mind that our approach has limitations. One of them is the complex relationship between co-expression and co-regulation. Common regulation of gene expression may result in correlated expression, but also in expression anticorrelation, or expression correlation with a time- or dosage-dependent delay [39]. In our approach, we consider only positive expression correlation, although the NeMo approach also includes positive correlation over a subset of conditions.

Using large sets of gene expression data, we have discovered patterns of co-expression of genes associated with different regions in the human genome, including those distant in sequence or located on different chromosomes. A cluster of co-expressed genes is typically spread on a lower-than-expected number of chromosomes. Also, co-expressed positional gene clusters are observed. They most often contain 2-3 genes. In the same co-expression cluster, more than one such positional cluster is often found. Decreased distances between genes from one chromosome in the same co-expression cluster were observed. Positional clusters in co-expression clusters come from places in the genome of varied local gene density. However, the local gene densities of genes from positional clusters within a co-expression cluster vary less than the local gene densities of genes from all positional clusters. Genes from the same co-expression cluster show a local gene density similarity that is not a result of positioning in the same region of the genome.

Some pairs of chromosomes often appear together in one co-expression cluster. The pairs are not identical in terms of the different approaches of obtaining transcription modules. For the NeMo dataset, not only pairs but also groups of chromosomes with co-expressed genes were recognised. These are chromosomes 9, 12, 15, 17, 20, and 22. The genome was also investigated in the context of expression with a higher resolution. Pairs of 10 Mbp regions associated with genes that are often co-expressed were found. Some, but a minority of such significant pairs of regions are identical for both datasets. Some 10 Mbp regions were recognised to contain genes that co-express with genes from many different regions in the genome and from various chromosomes. The regions that have many co-expressed partner regions are not spread among all chromosomes. In both datasets, they are located on chromosomes 1, 3, 4, 5, 9, 14, 15, 20, but for both datasets they are not the same regions. In some cases they have similar chromosome positions. Functional annotations elucidated for the groups of genomic regions associated with co-expressed genes point to biological processes that may underlie the long-distance expression correlations observed in this study. The sets of functional annotations enriched in co-expressed genes from groups of such genomic regions differ markedly between the two datasets analysed. For the NeMo dataset, which includes very diverse cell types and very diverse perturbations, functional terms appear that are related to basic cellular functions and nuclear protein genes, e.g. cell cycle. For the Symatlas dataset, which consists of tissue atlas data, functional terms appear that are related to extracellular functions and extracellular protein genes, e.g. cell-cell signalling. In general, co-expression may often be related to a particular cell type and perturbation. In this study, the long-distance co-expression relations for genomic regions elucidated are probably robust enough to appear despite the expression correlation signals being ‘diluted’ as a consequence of various conditions and cell types. Thus, the most notable long-distance co-expression among various normal tissues is related to between-tissue differences in cell-cell signalling, while the most notable long-distance co-expression among datasets including various external stimuli is related to basic cell cycle regulatory functions. The over-representation of groups of positional clusters in co-expression clusters suggests that the co-expression clusters observed may be in part related to chromosome territory contacts.

## Conclusions

Using simple permutation tests, we have shown that long-distance gene co-expression relationships can be elucidated that may be functionally relevant. Such relationships may or may not be related to the nucleus structure, yet also other factors and phenomena may be the underlying reasons. Comparison with experimental data on the three-dimensional structure of the nucleus that are beginning to become available [40] will enable an answer to be found to the question as to whether such long-distance gene co-expression is directly related to nucleus structure.

## Methods

### Co-expression clusters (transcription modules)

1. NeMo data: The first group of co-expression clusters used in this study was derived by Yan et al. [37] by means of a graph-based method [37]. The authors applied their graph-based method to 105 human microarray datasets and identified 4727 potential transcription modules, activated under different subsets of conditions (see Additional file 7). The clusters together contain 716 genes (see Additional file 8). The number of genes in clusters ranges from 7 to 20 (average 10.7 with a standard deviation of 2.9). The high quality of clusters was supported by transcription factor binding ChIP-chip experiments, analysis of putative transcription factor binding sites and functional homogeneity analysis [37].

2. Symatlas data: We also used an alternative way of identification of co-expression clusters. The dataset results from an experiment conducted on 79 human samples from different tissues, organs and cell lines [38]. This gene atlas represents the normal transcriptome. The gene expression profiles were clustered by means of agglomerative hierarchical clustering with the average linkage method (UPGMA). Pearson Correlation was used as a distance measure. The clusters were obtained by cutting trees at a level closest to leaves while maximizing the number of clusters containing between 7 and 30 genes. Thus, cluster sizes were kept comparable with the sizes of clusters from the NeMo approach. The upper limit of the cluster size was set at 30 genes in order to increase the number of clusters obtained (see Additional file 9). The Symatlas clusters together contain 2578 genes (see Additional file 10). We have also tried to obtain clusters by hierarchical clustering with single and with complete linkage algorithms. In both cases, only very few clusters of size between 7 and 30 genes were obtained (2 and 8 respectively), and consequently these last algorithms were not used here.

### Positional clusters

The genomic positions of genes in each co-expression cluster were clustered by average-linkage hierarchical clustering method. The genes more distant than 1 Mbp were considered as belonging to separate positional clusters.

### Groups of positional clusters

By a group of positional clusters we call more than one positional gene cluster found within one co-expression cluster.

### Randomised samples, significance estimation

For each gene in a co-expression cluster, we chose a random genomic position from among the positions of all such genes. We determined the statistical significance of a value by permutation analysis (10000 permutations), using a Z-score threshold of 3. Since the distributions analysed need not be normal, P-values were also calculated from the permutations with a significance threshold of 0.05 for each tail analysis.

### Randomisation of positional clusters

Each co-expression cluster was considered as a group of positional clusters and positionally separate genes that could not be assigned to a positional cluster. The number of such items (separate genes and positional clusters) in all co-expression clusters was kept constant but items were assigned to the co-expression clusters randomly (10000 times). After each randomisation, the number of groups of positional clusters within co-expression clusters was counted.

### Gene density, genomic windows

Gene density was calculated as the number of genes within a genomic region, divided by the length of the genomic region [Mbp]. Local gene density was calculated over a 1 Mbp window size, and overall gene density over whole chromosome length. Chromosome lengths and gene content were determined based on NCBI Build36.2 (see Additional file 2). For certain analyses, chromosomes were divided into non-overlapping 10 Mbp genomic regions, starting from the beginning of each chromosome.

### Co-expressed genes from pairs of genomic regions

We considered two types of the genomic regions: whole chromosomes and 10 Mbp-wide chromosome fragments and used them in separate analyses. For each pair of genomic regions, we counted the number of gene pairs from those regions (with each gene coming from a different region) that occur in one co-expression cluster. The measure of significance for a pair of regions is the difference between the gene pair count for real and randomised data (see Randomised samples section above) expressed as the number of standard deviations.

### Clusters of chromosomes associated with co-expressed genes

UPGMA (Unweighted Pair Group Method with Arithmetic Mean), and complete and single linkage hierarchical clustering was performed. The similarity measure was calculated as 1 – (x + |min(x)|)/(|max(x)| + |min(x)|) + eps; eps = 0.1; where x is the difference in the number of occurrences of a pair of chromosomes in the same co-expression cluster between real and randomised data, expressed as the number of standard deviations. The cut-off used is 3 standard deviations. The clusters below the cut-off obtained for the Symatlas data using complete, average and single linkage clustering are the same. In clustering of the NeMo data, the four chromosomes (9, 15, 17, and 20) in the cluster below the cut-off are the same for the three types of linkage. Chromosomes 12 and 22 are slightly above the cut-off only in complete linkage clustering. In single linkage clustering, besides chromosomes 9, 12, 15, 17, 20, 22, chromosomes 4, 7, 10 are also below the cut-off.

### Functional analysis

We used DAVID 6.7b tool [41] to analyse the significance of frequent occurrences of several categories of functional annotation terms in sets of genes. The following functional term categories were explored: OMIM DISEASE, SP PIR KEYWORDS, GOTERM BP ALL, GOTERM CC ALL, GOTERM MF ALL, KEGG PATHWAY, EC NUMBER, PFAM. The measure of significance of a term is the p-value with Bonferroni correction for multiple tests, the p-value cut-off used was 0.05. Terms are considered if they are represented by at least two genes. As the background gene set to the analysis, all the genes from the co-expression clusters for a given expression dataset were used.

### Scripts

For all the analyses, dedicated Python scripts were written, unless otherwise stated.

## Competing interests

The authors declare that they have no competing interests.

## Authors’ contributions

TS and KP conceived of the study, designed it, and drafted the manuscript. TS wrote the scripts and performed the analyses. Both authors read and approved the final manuscript.

## Supplementary Material

Additional file 1 **Average linkage hierarchical clustering trees of chromosomes that contain genes often co-expressed with each other.** Similarity measure depends on the number of occurrences of genes from a pair of chromosomes in the same co-expression cluster between non-randomised and randomised data, expressed as the number of standard deviations (see Methods). Clusters above the cut-off that equals three standard deviations are shown in red. Click here for file

Additional file 2 **Chromosome gene density table.** Chromosomes ordered by overall gene density. Achrocentric chromosomes marked. Click here for file

Additional file 3 **Regions with the highest number of co-expression partners.** Top 4% of regions (for different significance cut-off) that have the highest number of partners. Partnering regions are those pairs of regions from which genes are significantly often co-expressed. Click here for file

Additional file 4 **Functional annotations of genes from selected regions.** Significant functional annotation terms connected to genes from regions 81 - 89 (chr 4, q22.1-q34.1)), 233 (chr 14, q21.3) and 283 - 287 (chr 20, p12.3-q13.13) and their partnering regions. Genes from each region considered, together with genes from its partnering regions are separate queries in functional term enrichment analysis (see Methods). Click here for file

Additional file 5 **Functional annotation of genes from all significant pairs of regions together.** Significant functional annotation terms connected to genes from significant pairs of regions for Symatlas and NeMo data. Click here for file

Additional file 6 **Functional annotation of genes from groups of positional clusters in the same co-expression cluster.** The query gene set included genes from all such groups taken together for Symatlas data, and for NeMo data separately. Click here for file

Additional file 7 **NeMo co-expression clusters.** Each row includes Entrez Gene Identifiers of genes belonging to the same co-expression cluster. Click here for file

Additional file 8 **NeMo genes.** All genes from the NeMo co-expression clusters used. Entrez Gene ID, 10 Mb region number, chromosome number information. Click here for file

Additional file 9 **Symatlas co-expression clusters.** Each row includes Entrez Gene Identifiers of genes belonging to the same co-expression cluster. Click here for file

Additional file 10 **Symatlas genes.** All genes from the Symatlas co-expression clusters used. Entrez Gene ID, 10 Mb region number, chromosome number information. Click here for file
