# Supplementary figures and images for: Genomic positions of co-expressed genes: echoes of chromosome organisation in gene expression data
Source: BMC Res Notes. 2013 Jun 13;6:229. doi: 10.1186/1756-0500-6-229 (PMC3689077; doi:10.1186/1756-0500-6-229)

SymAtlas

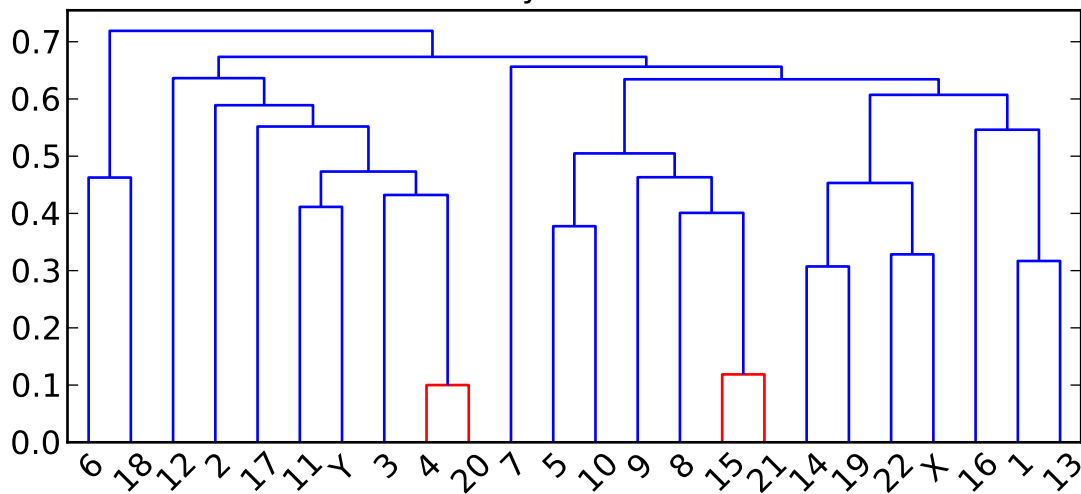

NeMo

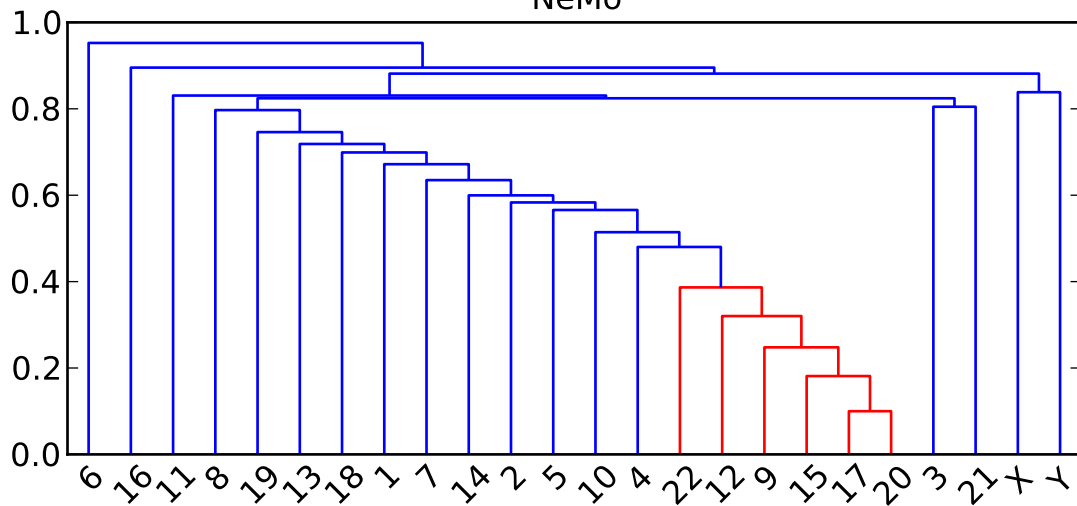

Supplement: Additional file 1 — Average linkage hierarchical clustering trees of chromosomes that contain genes often co-expressed with each other. Similarity measure depends on the number of occurrences of genes from a pair of chromosomes in the same co-expression cluster between non-randomised and randomised data, expressed as the number of standard deviations (see Methods). Clusters above the cut-off that equals three standard deviations are shown in red. [file 1756-0500-6-229-S1.pdf]
