# Supplementary material for: Genomic positions of co-expressed genes: echoes of chromosome organisation in gene expression data
Source: BMC Res Notes. 2013 Jun 13;6:229. doi: 10.1186/1756-0500-6-229 (PMC3689077; doi:10.1186/1756-0500-6-229)
Supplement: Additional file 2 — Chromosome gene density table. Chromosomes ordered by overall gene density. Achrocentric chromosomes marked. [file 1756-0500-6-229-S2.pdf]

Table. Chromosomes ordered by overall gene density

Chrom Nr	lengh [Mb]	Nr of genes	density	acrocentric
19	64	1695	26,48	
17	79	1469	18,59	
22	50	742	14,84	+
11	134	1848	13,79	
16	89	1109	12,46	
14	106	1275	12,03	+
20	62	737	11,89	
1	247	2782	11,26	
12	132	1370	10,38	
15	100	945	9,45	+
7	159	1452	9,13	
6	171	1505	8,80	
23	155	1336	8,62	
9	140	1148	8,20	
10	135	1106	8,19	
2	243	1888	7,77	
21	47	352	7,49	+
3	200	1469	7,35	
5	181	1268	7,01	
8	146	984	6,74	
4	191	1154	6,04	
18	76	432	5,68	
24	58	307	5,29	
13	114	551	4,83	+
