# Supplementary material for: Genomic positions of co-expressed genes: echoes of chromosome organisation in gene expression data
Source: BMC Res Notes. 2013 Jun 13;6:229. doi: 10.1186/1756-0500-6-229 (PMC3689077; doi:10.1186/1756-0500-6-229)
Supplement: Additional file 3 — Regions with the highest number of co-expression partners. Top 4% of regions (for different significance cut-off) that have the highest number of partners. Partnering regions are those pairs of regions from which genes are significantly often co-expressed. [file 1756-0500-6-229-S3.pdf]

Table. Top 4% of regions (for different significance cutoff) that have the highest number of partners.

	Symatlas		Nemo	
Chr.	Cutoff 3SD	Cutoff 5SD	Cutoff 3SD	Cutoff 5SD
1	7 (p31.3),<br>9 (p22.2, p22.3, p31.1)			23 (q42.12)
2	40 (q22.2, q22.3),<br>45 (q32.3, q33.1)	40 (q22.2, q22.3),<br>45 (q32.3, q33.1)		
3	59 (p12.2)	59 (p12.2)	67 (q25.33)	
4	78 (q13.1, q13.2),<br>87 (q31.3, q31.23, q32.1)	81 (q22.1,q23),<br>87 (q31.3, q31.23, q32.1)	82 (q23,q24),<br>84 (q27, q28.2),<br>89 (q34.1)	82 (q23,q24),<br>84 (q27, q28.2)
5	94 (p14.1, p14.2)	94 (p14.1, p14.2)	104 (q23.2)	104 (q23.2)
6		119 (q14.2, q14.3, q15)		
7				
8				
9		161 (p23)		169 (q22.2, q22.33)
10			180 (q21.1)	180 (q21.1)
11		197 (q14.1, q14.2, q14.3),<br>202 (q25)		
12				207 (q13.11, q13.12)
13	224 (q22.1, q22.3)			
14	233 (q21.3)	233 (q21.3)	233 (q21.3)	
15			243 (q13.3, q15.1),<br>246 (q22.31, q23)	243 (q13.3, q15.1)
16				
17				
18			268 (p11.32)	267 (q25.1, q25.3)
19				
20	284 (p11.23, p12.1),<br>287 (q13.12, q13.13)	284 (p11.23, p12.1)	285 (p11.21, q11.21),<br>287 (q13.12, q13.13)	283 (p12.3),<br>285(p11.21, q11.21),<br>287 (q13.12, q13.13)
21				
22			296 (q11.21)	296 (q11.21)
X				
Y		317 (p22.33)		
